# Epidemiological review on the resurgence of measles outbreaks in Canada during the post-elimination era: A scoping review

**DOI:** 10.1371/journal.pgph.0006295

**Published:** 2026-04-13

**Authors:** Ainrisq A. Rifai, Dana C. Pittman Ratterree, Jude D. Kong, Martial L. Ndeffo-Mbah

**Affiliations:** 1 Department of Veterinary Integrative Biosciences, College of Veterinary Medicine and Biomedical Sciences, Texas A&M University, College Station, Texas, United States of America; 2 Artificial Intelligence & Mathematical Modeling Lab, Dalla Lana School of Public Health, University of Toronto, Toronto, Ontario, Canada; 3 Department of Epidemiology and Biostatistics, School of Public Health, Texas A&M University, College Station, Texas, United States of America; New York University Grossman School of Medicine, UNITED STATES OF AMERICA

## Abstract

In 1998, the World Health Organization declared that measles had been eliminated in Canada. Despite this status, Canada continues to experience sporadic outbreaks driven by importations from endemic countries. Understanding the post-elimination outbreaks is crucial for informing prevention and control policies. The purpose of this review was to identify and analyze epidemiological factors of measles outbreaks’ resurgence in Canada. We conducted a PRISMA scoping review to compile epidemiological data from peer-reviewed articles and grey literature on outbreaks in Canada from 1999 to 2025. A total of 618 articles were retrieved from the 9 databases queried, and 144 grey literature items were collected in the manual search; including 18 peer-reviewed articles and 28 grey literature sources. Eighty distinct outbreaks with a total of 7,455 cases were reported between 1999 and 2025 where 61.7% of the outbreaks were associated with imported cases of measles and 79.7% of the cases had not received the Measles-Mumps-Rubella vaccine. More than 60% of the cases (5078) were related to the 2025 multijurisdictional outbreak. Post-elimination measles outbreaks in Canada are primarily caused by importation of the disease and subsequent transmission in local under-vaccinated communities.

## Introduction

Measles, or rubeola, is a highly contagious, acute febrile illness caused by a single-stranded, negative-sense RNA virus of the *Paramyxoviridae* family [1,2]. The virus is exclusively human in origin, with no animal reservoir. Transmission occurs primarily via respiratory droplets, aerosols, or direct contact. Measles is largely preventable through vaccination. The Measles-Mumps-Rubella (MMR) vaccine has a long-standing record of safety and efficacy, offering approximately 97% protection after two doses [1]. However, secondary MMR vaccine failure has been observed in approximately 5% of recipients after 6–26 years due to waning immunity [3]. The vaccination threshold for preventing community transmission is 95% [2]. Despite the availability of a safe and effective vaccine, measles remains a significant global public health concern, especially in regions with lower vaccination coverage [2]. Before 2020, Canada’s national MMR vaccine coverage was estimated to be around 90% among school-age children, but with high variability between regions [4,5]. For example, MMR vaccine coverage among school children was shown to vary between 45% to 95% across Alberta, and 75% to 92% in British Columbia [4]. This vaccination coverage is lower than the average MMR vaccine coverage in high-income countries and higher than that of most countries in the Americas [6].

The World Health Organization (WHO) grants measles elimination status to countries that have successfully mitigated sustained transmission [7]. The criteria for achieving this status include sustained interruption of endemic transmission, robust viral surveillance, high-quality immunization coverage, and sensitive case detection systems [7,8]. In Canada, measles was declared eliminated in 1998, yet sporadic outbreaks continue to emerge, largely due to importation from endemic regions [9]. Canada reported an average of 91 cases annually between 1998 and 2024 [10]. Canada defines a confirmed case as one with laboratory-confirmed diagnoses (RNA detection, virus isolation, IgG seroconversion, or IgM positivity) or as a clinical case with epidemiological links to confirmed infections [9].

Understanding epidemiological commonalities from post-elimination measles outbreaks in Canada provides context that can aid in preventing future outbreaks. The purpose of this scoping review was to systematically collect original research and grey literature describing the epidemiology of Canadian measles outbreaks post-1998 elimination declaration.

## Materials and methods

In this study, we conducted a scoping review to identify and analyze epidemiological factors contributing to the resurgence of measles in Canada. This was done by systematically compiling epidemiological studies and grey literature on post-elimination measles outbreaks in Canada. We examined outbreak characteristics, case demographics, transmission patterns, vaccination status, case importation sources, geographical spread, and case hospitalization rates. We highlight the insights that outbreak investigations provide on the role of international travel in seeding outbreaks of measles in under-vaccinated communities in Canada. The national public health guidelines for Canada define a measles outbreak as: (1) two or more epidemiologically or virologically linked cases with at least one laboratory confirmed case, or (2) one case with an unknown source of infection [11]. A laboratory-confirmed case is defined as: (1) positive serological test for IgM antibody, (2) seroconversion or greater than fourfold rise in IgG titre, (3) PCR detection of measles RNA in a clinical specimen, (4) isolation of the virus from a clinical specimen [11]. Clinical cases are defined as having a generalized maculopapular rash, fever, and one or more of cough, coryza, or conjunctivitis [11]. An epidemiological linkage is defined as a case that has exposure as: (1) a setting or community experiencing an outbreak of measles, (2) contact with one or more cases, (3) a geographic area where measles is endemic or an outbreak of measles is occurring [12].

### Ethical considerations

An ethics statement is not applicable because this systematic review is based exclusively on published literature. Anonymity of all subjects is ensured due to the design of the review.

### Search strategy and selection criteria

This study used the Preferred Reporting Items for Systematic reviews and Meta-Analyses - Scoping Reviews (PRISMA-ScR) guidelines [13]. The search was restricted to articles published between January 1, 1999, and July 21, 2025. Searches were conducted in PubMed, MEDLINE Complete, CAB Abstracts, EMBASE, Web of Science, CINAHL, ProQuest, and Global Index Medicus. Table 1 presents the search terms by category used to identify literature. Full search strings for each database are in the supplementary materials.

**Table 1:** Search terms by category.

Category	Terms
**Disease**	Measles, Measles virus, Rubeola, Red measles, English measles, Mononegavirales Infections, Paramyxoviridae Infections, Morbillivirus Infections
**Study Design**	Epidemiology, Contact Tracing, Epidemiologic, Disease Outbreak, Disease Outbreaks, Outbreak, Outbreaks, Infectious Disease Outbreaks, Epidemic, Epidemiologic Methods, Data Collection, Population Surveillance, Epidemiological Monitoring, Community outbreak, Outbreak Investigation, Retrospective study, Transmission, Epidemic, Surveillance
**Location**	North America, Canada

To be included in this review, the paper has to be a descriptive observational epidemiology report on individuals naturally exposed to the measles virus and meet the definition of a measles outbreak for Canada. Studies on sporadic cases, non-outbreak events, or outbreaks reported before 1998 were excluded. Additionally, study designs such as clinical trials, laboratory-based studies, vaccine efficacy trials, or experimental interventions that did not report real-world outbreak data were excluded. Studies conducted outside of Canada or at the global level that did not have granular data about Canada were also excluded. Lastly, studies without original data or published in a language other than English or French (without a reliable English translation) were excluded.

### Grey literature search

The purpose of the grey literature search was to supplement data not captured in indexed literature. A manual Google search was used to identify outbreak investigations and governmental reports from national and regional health entities, and news articles from reputable sources. The initial search was restricted to materials published between January 1, 1999, and July 21, 2025, using the same inclusion/exclusion criteria as the peer-reviewed literature. To account for the one-year mark of the ongoing measles outbreak in Canada, a second search of the grey literature was conducted on October 15, 2025, to capture newly reported cases.

### Article selection and data extraction

After searching the databases, records were imported into Covidence software, a web-based collaboration software platform that streamlines the production of literature reviews (Veritas Health Innovation, Melbourne, Australia). Duplicates were automatically flagged by Covidence and manually confirmed by one reviewer (AR). The selection of reports was conducted in two phases. Phase one was title and abstract screening, and phase two was full-text review; both phases were conducted by two reviewers (AR & DPR). All consensus on discrepancies was achieved through discussion between AR and DPR.

Study characteristics were tabulated for outbreak year, province, health territory or public health unit, index case(s), mode of transmission, genotype (strain), total number of cases, and MMR vaccination status among cases. Separate tables were developed to synthesize data for demographic characteristics such as age, sex, outbreak setting, and importation status across all the years of interest. A χ^2^ Goodness-of-Fit test was performed to determine if cases were equally distributed across age groups or provinces. Standardized residuals were used to measure the strength of the difference between the observed and expected case counts.

A meta-analysis was conducted using all identified literature reporting hospitalization status to estimate the pooled proportion of hospitalized cases. Pooled estimates were calculated using a random-effects model with inverse variance weighting. The I-squared statistic was used to quantify heterogeneity (I²), and the restricted maximum-likelihood (REML) estimator was used to estimate between-outbreak variance (τ²). To explore sources of heterogeneity, we conducted subgroup analyses by year of study, sample size, and weight in the meta-analysis. Meta-regression was not performed due to the limited number of studies. Publication bias was assessed graphically using a funnel plot and statistically by the Egger test. The analysis was performed in RStudio (version 2024.12.1 + 563; Posit Software, PBC, Boston, MA) using the “meta” [14], “metadat” [15], and “metafor” [16] packages. The pooled estimates were computed using the *metaprop()* function, the Hartung-Knapp adjustment (df = 7) was applied with a logit transformation for proportion estimates, and results were visualized as forest plots generated by the *forest()* function available within the meta package.

To determine how the distribution of measles across Canada changed over time, outbreak cases and health region data were divided into 1999–2023 and 2024–2025 multijurisdictional outbreaks. Shapefiles for Canadian health regions were acquired from Statistics Canada, a government data repository [17]. Census data for each province between 2006 and 2021 was also acquired from Statistics Canada [18,19]. The R package “tmap” [20] was used to generate a heat map of cases by health region in RStudio (version: 2025.09.0 + 387 Posit team (2025). RStudio: Integrated Development Environment for R. Posit Software, PBC, Boston, MA.).

## Results

**Fig 1:**
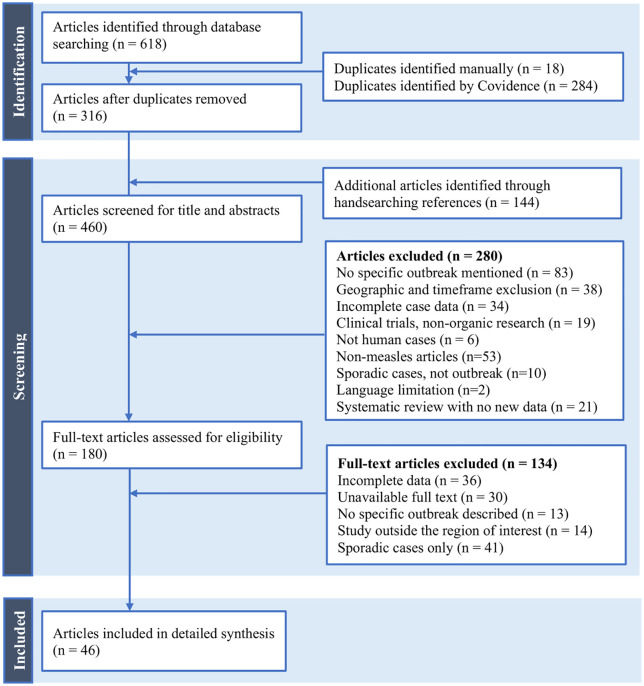
PRISMA-ScR flow diagram of record identification, screening, eligibility, and inclusion.

In this review, 618 articles were retrieved from the 9 databases queried, and 144 grey literature items were collected in the manual search (Fig 1). After removing duplicate records, the titles and abstracts were screened for the remaining 460 articles. There were 180 records eligible for full-text screening, of which 18 peer-reviewed articles and 28 grey literature sources underwent data extraction (S1 Table).

### Outbreak characteristics

Between January 1999 and October 2025, 80 distinct measles outbreaks were identified involving 7,455 reported cases (S2 Table). Measles outbreaks were considered distinct if they were identified as such in the literature. This was generally based on the lack of an epidemiological link between outbreaks (S2 Table). Frequent outbreak settings included high schools, healthcare facilities, childcare centers, and workplaces. The most recent and largest outbreak started in October 2024 and has resulted in 5,078 cases as of October 15, 2025 (Table 2). This outbreak was linked to cases from New Brunswick [21]. Before the multi-jurisdictional outbreaks of 2024/2025, Canada had experienced two large-scale outbreaks. Besides these multijurisdictional events, measles outbreaks have been sporadic, with years where low or no outbreak-related cases were reported. The first large-scale outbreak occurred in 2011 with 791 cases, primarily from Quebec and British Columbia [22,23]. The second outbreak occurred in 2014 with 520 cases from transmission events primarily in British Columbia. Multijurisdictional outbreaks were assigned to provinces based on case distributions reported in the literature.

**Table 2:** Characteristics of post-elimination measles outbreaks.

	2011	2014	2024	2025	Other Years	All Years
**Total Cases**	**791**	**520**	**197**	**5078**	**869**	**7455**
Laboratory Confirmed	301	68	50	407	826	925
Epidemiologically Linked	431	322	1	4289	68	5111
Clinical Cases	58	110	0	335	0	503
Unknown	1	20	146	47	214	916
**Vaccination Status**	**384**	**465**	**146**	**4590**	**564**	**6149**
2 doses	80	4	12	200	135	431
1 dose	90	7	14	109	94	314
Unvaccinated	101	398	97	4096	206	4898
Unknown	194	56	23	185	48	506
**Sex**	**19**	**474**	**146**	**2374**	**236**	**3249**
Male	11	239	82	1236	114	1682
Female	8	235	64	1138	122	1567
**Age group (years)**	**744**	**474**	**146**	**4582**	**249**	**6195**
Age < 1	24	15	11	157	10	217
Age 1–4	63	75	24	1041	52	1203
Age 5–9	61	129	44	688	41	963
Age 10–14	255	176	0	1464	55	1950
Age 15–19	226	61	0	41	39	367
Age 20–29	45	14	65	486	31	641
Age 30–39	56	1	0	549	31	638
Age > 40	14	3	2	156	11	189
**Importation Status**	**4**	**21**	**3**	**2**	**54**	**84**
Imported	3	12	1	1	40	57
Non-imported	2	0	2	1	3	8
Unknown exposure	0	9	0	0	10	19

The characteristics and demographics of the outbreaks by year were compiled in Table 2. Overall, 84.01% of cases had no history of MMR vaccination (n = 4,692), 8.20% had no known vaccination record (n = 458), 2.49% had received one dose (n = 139), and 5.30% had received both doses (n = 296). Domestically, MMR vaccine refusal for religious or philosophical reasons has facilitated measles outbreaks and local transmission events, particularly within suboptimal vaccinated communities with transnational ties. This pattern of vaccine refusal facilitating the spread of measles was also evident in numerous other outbreaks across Alberta, British Columbia, and Quebec [24–31]. Age group information was categorized for 5,946 cases (16 out of 80 outbreaks) and the remaining reports only provided median or age range data. Case distribution was not uniform across the age groups (χ^2^(df = 7) = 3186.7, p < 0.001), the 10–14 age group (standard residual = 45.39) had a significantly higher number of cases than other age-groups (S3 Table). The highest proportion of cases occurred in children 10–14 years (n = 1,895; 31.87%), followed by toddlers ages 1–4 years (n = 1,203; 20.23%) (S3 Table). Adults 40 years and older and infants younger than a year old had the lowest proportion of reported cases (S3 Table).

The duration of reported measles outbreaks in Canada varied widely in the 25 outbreaks that recorded this data. The duration ranged from 4 to 349 days (1–50 weeks) with a median of 45 days (6 weeks). The shortest recorded outbreak involved a single generation of transmission in Alberta in 2014 [32]. The longest documented outbreak spanned nearly 50 weeks in Quebec, lasting from January 8 to December 22, 2011 [30]. The ongoing 2024–2025 Canadian measles outbreak, which as of October 15, 2025, has persisted for 51 weeks (356 days) is the longest outbreak duration; however, this figure is not included in the median calculation and is subject to change with future updates [21].

#### Imported cases.

Imported cases have been the primary drivers of outbreaks in Canada. Canadian public health defines an imported case as infections acquired abroad during the incubation period, supported by epidemiological or virological evidence [11]. Analysis of outbreak transmission events revealed that 57 outbreaks were imported by travelers who subsequently spread the infection locally. Importations from France (826 cases across 2 outbreaks) and the Netherlands (496 cases across 3 outbreaks) accounted for 62.47% of associated cases (Table 3). The 2013 Alberta outbreak, for instance, was linked to an ongoing European epidemic dominated by genotype D8 [33]. Out of the 57 imported outbreaks, 10 (17.5%) originated from the Philippines, resulting in a total of 91 associated cases; 5 (8.8%) originated from India, resulting in 10 associated cases; and 5 (8.8%) outbreaks originated from the United States, resulting in 172 associated cases. Two of the outbreaks implicating the United States were related to unvaccinated children attending well-known theme parks [32,34,35]. For example, the 2015 Quebec (Lanaudière region) outbreak was traced to a traveler exposed at a California theme park, resulting in 159 cases in the province [32,35,36].

**Table 3:** Country of importation in 57 of import-related outbreaks.

Country of Origin	# of Imported Outbreaks (%)	Location in Canada	# of Associated Cases	Genotype(s)
**Europe**				
Europe, unspecified	3 (5.3%)	Alberta, New Brunswick, Prince Edward Island, Saskatchewan	20	D8
Netherlands	3 (5.3%)	Alberta, British Columbia	496	D6, D8
Germany	1 (1.8%)	Alberta	3	D5
France	2 (3.5%)	Quebec	826	B3, D4
United Kingdom	1 (1.8%)	New Brunswick, Ontario	4	D8
Ukraine	1 (1.8%)	Ontario	2	D8
Romania	1 (1.8%)	Quebec	4	B3
**Americas**				
Mexico	1 (1.8%)	Alberta	6	D7
Bolivia	1 (1.8%)	Alberta, British Columbia	155	D6
United States	5 (8.8%)	British Columbia, Quebec, Ontario	172	B3, D4, D8
**East Asia**				
Korea	1 (1.8%)	British Columbia	8	H1
China	3 (5.3%)	British Columbia, Ontario	96	D9, H1
**Southeast Asia**				
Southeast Asia Unspecified	2 (3.5%)	British Columbia, Manitoba	79	D8
Philippines	10 (17.5%)	Alberta, British Columbia, Northwest Territories, Ontario, Saskatchewan	91	B3, D8
Thailand	2 (3.5%)	British Columbia, Ontario	4	B3, D8
Viet Nam	3 (5.3%)	Alberta, British Columbia	21	D8
Singapore	1 (1.8%)	Ontario	3	H1
**South Asia**				
Pakistan	2 (3.5%)	British Columbia	4	
India	5 (8.8%)	Alberta, British Columbia, Manitoba, Quebec	10	D4, D8
Bangladesh	1 (1.8%)	Ontario	2	B3
**Pacific Islands**				
New Zealand	2 (3.5%)	Alberta, British Columbia	5	D5
**Other**				
Unknown Country	2 (3.5%)	Alberta, British Columbia	16	
Suspect Importation	4 (7.0%)	British Columbia, Ontario	89	D6, D8
**Total Imported**	57 (100%)		2,116	

#### Superspreading events.

Superspreading events were another key driver of large outbreaks. These events typically involve extensive transmission following exposure at large social gatherings. The literature search identified three notable examples. The first event was in 2000, affecting the provinces of Alberta and British Columbia, resulting in 155 cases [24,25,26]. The index cases came from two Alberta families that were a part of a religious community that had traveled to Bolivia in May. In June, the measles-affected families attended a large social gathering in British Columbia that resulted in 4 British Columbia families contracting the disease and subsequently transmitting the disease to other families in the community [25]. Next, the 2010 Winter Olympic Games held in Vancouver, British Columbia resulted in 80 confirmed cases [37]. The Winter Olympics were held in February; however, the first 3 cases from adults who attended the event were not identified until March 9–11, with additional 2 cases later identified on March 19 [38]. The H1 and D8 genotypes were the strains that resulted in sustained transmission from March 9th through April 28th, primarily affecting those with no immunization or unknown vaccination history [37,38]. Most recently, the multijurisdictional outbreak of 2024–2025 is linked to a Mennonite Community gathering in New Brunswick [21,39]. The index case was an adult who traveled to Canada for this event in mid-October, and subsequent cases in early November were epidemiologically linked to individuals who had also attended [40].

### Hospitalization

Hospitalization data were inconsistently reported across the reviewed sources; between 1999 and 2025, hospitalization outcomes were available for 9 major measles outbreaks. Out of the 6,478 reported measles cases, only 489 resulted in hospitalizations. British Columbia’s 2014 outbreak resulted in 5 hospitalizations of 2 infants and 3 adults, with 1 case of encephalitis but no deaths. During the 2024–2025 Canadian multijurisdictional outbreak, two deaths have been recorded, one in Ontario and one in Alberta. One of the deaths involved a preterm newborn with congenital measles and underlying medical conditions [10]. While in Ontario, specifically in 2025, the outbreak became the largest in the Western Hemisphere, with 119 individuals hospitalized (including 9 in intensive care). Moreover, 40 pregnant women were infected during this outbreak, of whom only 2 had been vaccinated. Few deaths were documented in the reviewed outbreaks, and complications were more frequently reported. Overall, 27 patients were admitted to intensive care during the 2024–2025 Canadian multi-jurisdictional outbreak as of October 2025. In Quebec’s large 2011 outbreak, 8% (64 cases) developed complications. The most common complications were respiratory diseases, such as pneumonia, which was diagnosed in 42.8% (27 cases) of patients with complications [26–28,41]. During the 2016 Quebec outbreak, approximately 8.8% of cases developed pneumonia, along with two cases of bronchitis and four ear infections (2.5%) [32,35].

#### Meta-analysis of hospitalization rate.

**Fig 2:**
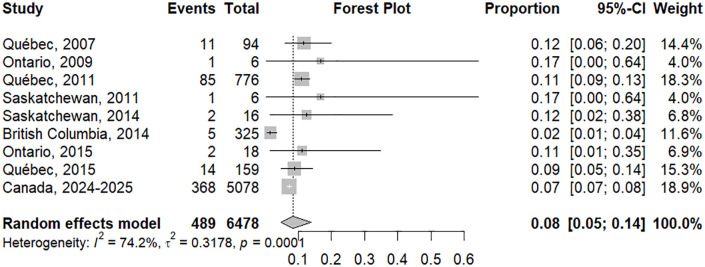
Forest plot of pooled hospitalization proportion among measles cases. Pooled estimates were calculated using a random-effects model with inverse variance weighting. The restricted maximum-likelihood (REML) estimator was used to estimate between-outbreak variance (τ²). The Hartung-Knapp adjustment (k = 9) was applied with a logit transformation for proportion estimates.

A total of nine studies involving 6,478 participants were included in our analysis. Our random-effects meta-analysis provided an estimated overall hospitalization pooled proportion of 8.0% (95% CI: 5.0-14.0) with substantial heterogeneity between the studies (I² = 74.2%, P < 0.0001) (Fig 2).

Subgroup analysis was conducted by study period, outbreak size, and study weight (S1 Appendix). Analysis by study period showed that outbreaks occurring before 2015 had a pooled hospitalization proportion of 9.0% (95% CI: 3.0%–21.0%), with substantial heterogeneity (I² = 75.8%, p < 0.001), and outbreaks reported after 2015 had a pooled hospitalization proportion of 7.0% (95% CI: 6.0%–8.0%) and no observed heterogeneity (I² = 0.0%, p = 0.63). When stratified by outbreak size, smaller outbreaks (<100 confirmed cases) had a pooled hospitalization proportion of 12.0% (95% CI: 10.0%–14.0%) with no observed heterogeneity (I² = 0.0%, p = 0.99), and larger outbreaks (≥100 confirmed cases) had a pooled hospitalization proportion of 6.0% (95% CI: 2.0%–21.0%), with considerable heterogeneity (I² = 88.9%, p < 0.0001). While stratification by study weight (weight in the meta-analysis) showed that lower-weight studies (<15%) yielded a pooled hospitalization proportion of 8.0% (95% CI: 3.0%–21.0%) with substantial heterogeneity (I² = 72.1%, p = 0.003), and higher-weight studies (>15%) demonstrated a pooled hospitalization proportion of 9.0% (95% CI: 5.0%–15.0%) and similarly high heterogeneity (I² = 84.6%, p = 0.0015).

#### Publication bias.

**Fig 3:**
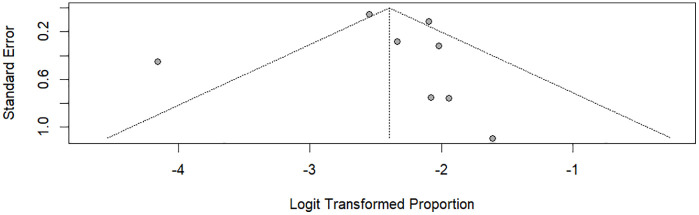
Funnel plot for assessment of publication bias.

Visual inspection of the funnel plot suggested some asymmetry; however, most studies fell within the confidence limits, and smaller studies were distributed on both sides of the pooled estimate. Visual asymmetry could not be reliably interpreted as evidence of publication bias, given the limited number of included studies (k = 9). Consistent with this observation, Egger’s regression test was not statistically significant (p = 0.6136); however, its interpretability was limited by low statistical power due to the small number of studies (Fig 3).

### Geographic distribution of cases

**Fig 4:**
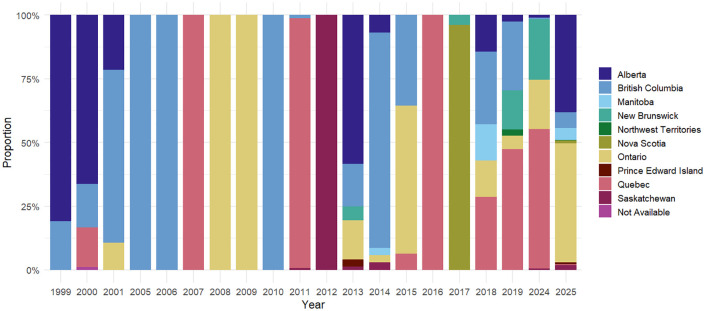
Distribution of measles cases by province/territory.

The geographic distribution of the 7,455 cases varied widely across the country, affecting 9 of 10 provinces and 1 of 3 territories (Fig 4, S4 Table). Notably, Newfoundland and Labrador, Yukon, and Nunavut have reported no outbreaks in the 46 sources reviewed. Conversely, British Columbia reported measles activity in 14 out of 26 years, suggesting persistent vulnerability to outbreaks [31]. Over the 26-year period, 33.9% (n = 2,526) of all measles cases were reported in Ontario, 29.1% (n = 2,170) were reported in Alberta, 16.4% (n = 1,225) were reported in Quebec, and 12.9% (n = 958) were reported in British Columbia. 94% of cases reported in Ontario and 89% of cases reported in Alberta occurred during the 2024–2025 multijurisdictional measles outbreak in Canada. 62% of cases reported in Quebec were from the 2011 outbreak (n = 755) [26]. Health unit-level geographic data were available for 7,082 cases (95.0%). Cases were categorized into two temporal periods, 1999–2023 and 2024–2025, and mapped at the health unit level (Fig 4, S1 Fig).

#### 1999-2023 Outbreaks.

**Fig 5:**
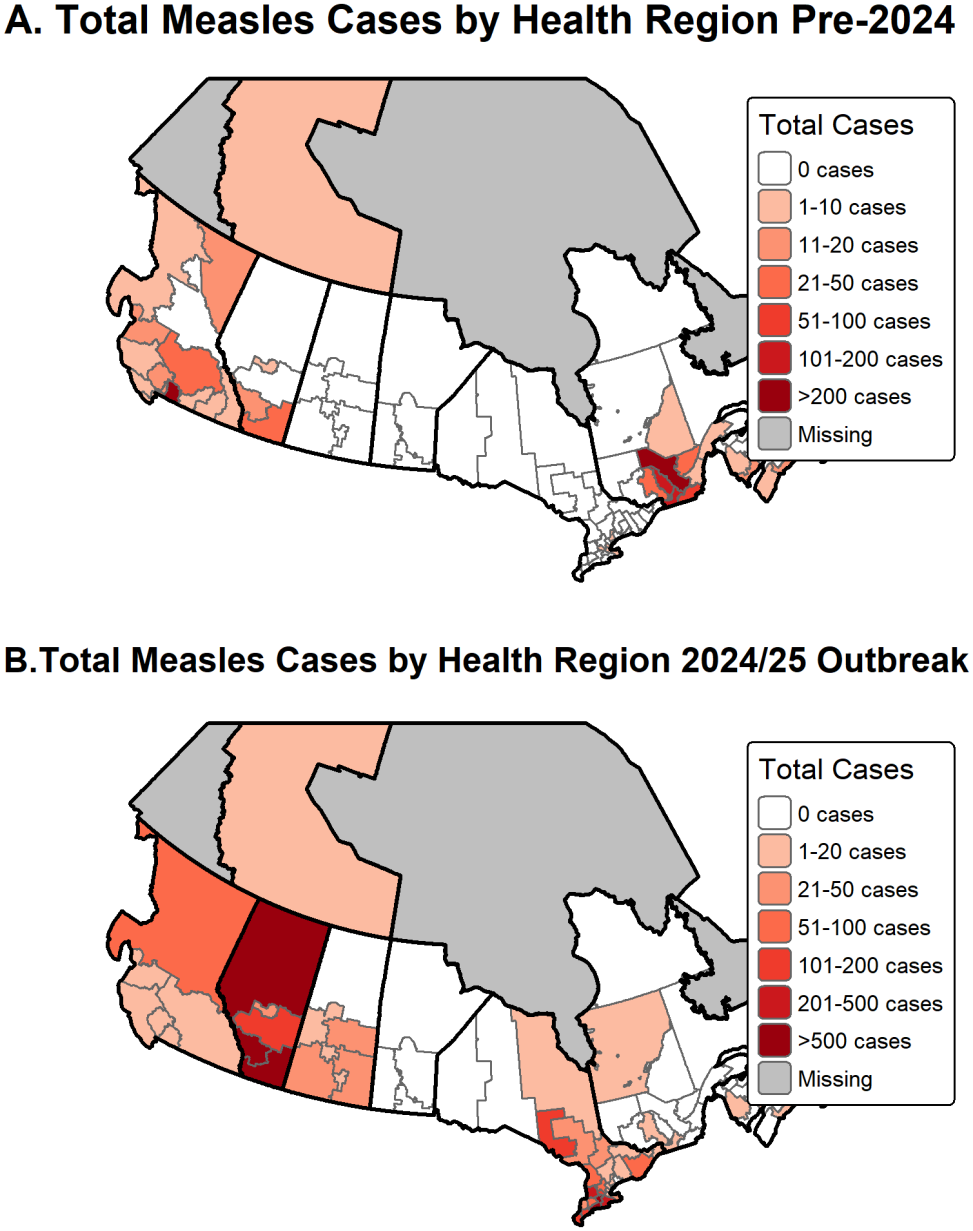
A) Total cases per health unit between 1999-2023 (n = 1,820). B) Total cases per health unit for the multijurisdictional outbreak 2024-2025 (n = 4,061). The shapefile for the Health Regions of Canada can be accessed at https://open.canada.ca/data/en/dataset/af595f3f-9f8c-4f69-bbb5-f740f0299c06/resource/429b7b52-b9b1-41ea-b16f-3770654723b4. https://open.canada.ca/en/open-government-licence-canada.

Between 1999 and 2023, 2,180 cases were reported in 9 provinces and 1 territory (Fig 4 and S4 Table). The geographic distribution of cases during this period deviated from population-weighted expectations (χ^2^(df = 12) = 3186.7, p < 0.001) indicating cases were concentrated in some provinces (S5 Table). The provinces of Quebec (n = 1,101, 50.5%, std. residual = 30.10) and Birstih Columbia (n = 634, 29.1%, std. residual = 21.99) accounted for the highest number of cases, while Ontario had significantly fewer cases than expected based on the population size (n = 113, 5.2%, std. residual = -31.77) (S5 Table). Of those 2,180 cases, there were 1,820 cases with health unit-level data reported in 39 health units in 6 provinces and 1 territory (Fig 5A). In Quebec, measles outbreaks were reported in 11 of the 18 health units, with the case count ranging from 2 to 546 cases. The Mauricie et Centre-du-Québec Health Unit reported 51.3% (n = 546) of cases, Montérégie reported 16.2% (n = 173), and Lanaudière reported 15.6% (n = 166) of cases. In British Columbia, 76.8% (n = 434) of cases occurred in the Fraser Health Unit. In Ontario reported, 56.6% (n = 64) of cases were reported from the City of Toronto Health Unit. Alberta reported 64 cases, New Brunswick reported 17 cases, and Nova Scotia reported 25 cases. No measles cases were reported in the provinces of Saskatchewan or Prince Edward Island during this period. Among the territories, 2 cases were reported in the Inuvik health region of the Northwest Territories.

#### Multijurisdictional outbreak.

Between 2024 and 2025, 5,275 measles cases were reported across 61 health units spanning 9 provinces and 1 territory (Fig 5B). The geographic distribution of cases during this period deviated from population-weighted expectations (χ^2^(df = 12) = 4392.9, p < 0.001) indicating cases were concentrated in specific provinces (S5 Table). Ontario and Alberta reported the highest number of cases, with 2,413 (45.74%) and 1,937 (36.72%) cases, respectively (S5 Table). Ontario had the highest case count, but Alberta was the most significant geographic outlier (std. residual = 57.33) indicating a disproportionate disease burden relative to its share of the national population. In Alberta, cases were mostly concentrated in the South Zone (n = 998, 18.92%), which includes the communities of Medicine Hat, Brooks, and Lethbridge. The North Zone reported 721 cases (13.67%), and the Central Zone reported 108 cases (2.05%). Notably, neither the North nor the Central zones had reported any measles cases before 2024. In Ontario, cases were reported in 33 out of 34 Public Health Units (PHU). The Oxford Elgin St. Thomas PHU reported 14.62% (n = 771) of cases, Grand Erie reported 5.67% (n = 299) of cases, and Huron Perth reported 5.63% (n = 297) of cases. The remaining PHUs reported between 1 and 179 cases each.

Both Quebec (std. residual = -35.61) and British Columbia (std. residual = -16.03) were significantly under-represented relative to their national population shares in the multijurisdictional outbreak. Quebec reported case data across four health units: Montréal (n = 24), Montérégie (n = 15), Centre-du-Québec (n = 13), and Laurentides (n = 4). British Columbia reported between 1 and 208 cases across the Northern, Island, Central, and Interior Health Authorities. Saskatchewan, which had not reported any health unit-level cases before 2024, reported 104 cases across seven of its health units. Prince Edward Island reported three cases, while Nova Scotia recorded one case in the Central Health Region. Manitoba, New Brunswick, and Prince Edward Island did not publicly release health unit-level data, though Manitoba reported a high overall case count during the 2024–2025 period (n = 242) based on provincial-level surveillance.

## Discussion

Canada has been regarded as a public health success story for controlling vaccine-preventable diseases since achieving measles elimination status in 1998; however, the constant importation of measles leading to periodic outbreaks highlights the fragility of this status. Our search identified 80 outbreaks involving 7,455 cases over the 26 years. Children aged 10–14 years accounted for the largest proportion of cases (31.5%), followed by toddlers aged 1–4 years (19.9%). No outbreak-related cases occurred between 2002–2004 and 2019–2023. The first outbreak-free period likely reflects the impact of mass vaccination campaigns. The more recent period is potentially related to the reduction in international travel and breakdown of public health surveillance systems during the COVID-19 pandemic. The 2025 multijurisdictional outbreak is the largest event since elimination was certified, contributing to over half the cases identified in this epidemiological review.

The MMR vaccine is highly effective, providing ~97% protection against measles after two doses [42]. The introduction of routine two-dose MMR vaccination and nationwide catch-up campaigns in the mid-1990s dramatically reduced measles incidence in the following years, allowing Canada to attain elimination status. Between 2002 and 2004, reported cases fell below 20 annually [25]. Effective vaccines, such as the MMR vaccine, require sustained coverage to prevent the accumulation of susceptible individuals necessary to fuel future outbreaks. The COVID-19 pandemic disrupted vaccine programs globally. Globally, more than 22 million infants missed their first dose in 2020 alone, the largest setback in two decades [43]. In Canada, MMR vaccine coverage for children aged two fell from 89.5% to 82.5% between 2019 and 2023 [44]. Additionally, 10–13% of children experienced a delay in receiving their 12-month dose [45]. The delay and reduction in vaccination places communities at risk of outbreaks when measles is reintroduced through travel. Furthermore, a modeling study suggests that even in communities with >90% vaccination coverage, outbreaks can occur if public health responses are delayed [4]. Importantly, sustaining elimination requires not only high coverage but also rapid identification and containment of new cases [4].

Our analysis revealed that the majority of outbreak-associated cases were unvaccinated. Religious objections, skepticism about medical interventions, and reliance on disease-induced herd immunity have contributed to under-vaccinated communities [46]. These clusters can serve as focal points for rapid transmission once measles is introduced. In New Brunswick, MMR vaccine coverage at age two declined from 87% in 2017 to 84.9% in 2019 [47,48]. During the 2011 outbreak, a qualitative analysis of public comments in Quebec showed a notable proportion expressing mistrust toward vaccines, citing concerns about safety, pharmaceutical companies, and personal freedoms [49]. The study found higher anti-vaccination sentiment in French-language media compared to English, with frequent concerns about children receiving too many vaccines [49].

Following measles elimination, various public health interventions were implemented in response to outbreaks. Primarily, vaccination campaigns were used to interrupt transmission. For example, during a 1999 outbreak in Alberta and British Columbia, reactive vaccination campaigns were implemented but yielded different degrees of success. Alberta was able to successfully interrupt transmission while British Columbia faced vaccination resistance [25,50,51]. Despite offering immunization and immunoprophylaxis, most individuals declined and were advised to avoid workplaces and public gatherings [25,50,51]. Subsequent outbreaks saw the widespread use of school-based vaccination programs. Notably, in Quebec, a province-wide campaign from November 2011 to June 2012 vaccinated over 34,000 students with their first dose [28]. The provincial vaccine registry indicated approximately 11% of students remained unvaccinated or lacked parental consent by 2013 [28]. During that outbreak, post-exposure prophylaxis with immunoglobulin was administered to high-risk groups, but large-scale supplementary vaccination and school exclusion policies were not widely deployed [27]. The 2014 outbreaks in Alberta and British Columbia highlighted both successes and challenges. Alberta#39;s baseline interventions included public communications and temporarily lowering the vaccination age from 12 to 6 months [52]. Additional measures, like drop-in and mass immunization clinics in Calgary, reduced outbreak duration to 44 days compared to 66 days in Edmonton [52]. In British Columbia, the response included MMR vaccine, immunoglobulin, private vaccination venues, school closures, travel restrictions, and engagement with religious leaders to address vaccine hesitancy [53].

Canada’s surveillance system has been instrumental in detecting imported and import-related cases. Federal surveillance depends on voluntary provincial and territorial reporting, but the Canadian Notifiable Disease Surveillance System (CNDSS) lacks the granularity required for elimination-level monitoring [9]. Global measles surveillance deteriorated during the COVID-19 pandemic, as the WHO and CDC reported the lowest laboratory specimen submissions in over a decade [43]. This interruption in surveillance could explain the lack of measles cases reported between 2020 and 2023, as public health resources were diverted to the pandemic. The interruption may have masked ongoing transmission in certain regions and delayed response efforts. Importation remains a fundamental driver of measles epidemiology in Canada. Among outbreaks with identified sources identified in this epidemiological review, 67.9% were linked to international travel. The 2011 Quebec outbreak illustrated how global connectivity influences domestic risk. Rising measles activity in France corresponded with increased cases in Quebec during that period, leading to the second largest outbreak during the 26 years of elimination [54]. When comparing neighboring provinces, despite Ontario being a major travel hub, there was higher immunity due to robust vaccine uptake among citizens and a larger proportion of immigrants with natural immunity [27]. On the other hand, Quebec’s lower vaccination coverage among students, coinciding with postulated superspreader events after spring break travel, contributed to extensive transmission [27].

The scoping review had limitations related to data completeness, consistency across sources, and underlying issues within the national surveillance infrastructure, which should be considered when interpreting the findings. Significant challenges arose due to the inconsistent reporting of key epidemiological factors across the identified sources. Data on hospitalization status were inconsistently reported, with records available for only 8 outbreaks between 1999 and 2025. Similarly, information regarding the outbreak duration was missing for most outbreaks, with information reported for only 25 out of 80 outbreaks reviewed. Additionally, data regarding categorized age groups were limited to 16 outbreaks, meaning that the remaining case reports only provided median or age range statistics. Furthermore, the study’s reliance on reported public health data was constrained by limitations inherent to Canada’s surveillance infrastructure.

Although Canada has not reported endemic transmission since 1998, large outbreaks involving imported cases challenge the sustainability of this status. The Pan American Health Organization’s criteria for elimination emphasize high vaccination coverage, sensitive surveillance, and prompt outbreak response [55]. Vaccination coverage is measured biennially at ages two and seven; coverage for individuals aged 1–40 years is not systematically assessed. The Measles and Rubella Surveillance Pilot Project reported national investigation rates for measles-like illness ranging from 12 per 100,000 population in 2006 (non-outbreak year) to 19 per 100,000 in 2011 (outbreak year) [48]. Canada’s inability to achieve 95% coverage in most childhood vaccinations [44,56] raises critical questions about whether the country can continue to meet these standards.

## Conclusion

Although Canada achieved measles elimination in 1998, the country has since reported 80 outbreaks involving 7455 cases. Outbreaks were largely driven by importation and subsequent spread in under-vaccinated communities. The ongoing multijurisdictional outbreak raises concerns about the sustainability of Canada’s elimination status, as more than 5078 cases were reported by October 15, 2025. Looking forward, maintaining measles elimination in Canada will require a multipronged approach. Strengthening routine immunization programs and addressing gaps in under-immunized communities are critical to restoring population immunity. Investing in targeted communication strategies to rebuild trust in vaccines is essential to achieve herd immunity, particularly in communities that have been affected by misinformation. Enhancing surveillance systems with more complete and timely reporting across provinces and territories to ensure rapid detection and response to imported cases. These efforts are especially urgent in light of the 2024–2025 multijurisdictional outbreak because it highlights how quickly Canada’s elimination achievements can be eroded. Without decisive action, the country risks losing its measles elimination status.

## Supporting information

S1 Appendix Study characteristics and publication bias assessments for the measles hospitalization meta-analysis. (PDF)

S1 Fig Reported measles cases by province, 1999–2025. (TIFF)

S1 Table Summary of measles outbreaks in Canada, 1999–2025. Outbreak data compiled through October 15, 2025.
(PDF)

S2 Table Characteristics of measles outbreaks in Canada by year, 1999–2025. Outbreak data compiled through October 15, 2025.
(PDF)

S3 Table Age group distribution of measles cases with standardized residuals. (PDF)

S4 Table Provincial distribution of measles cases in Canada by year, 1999–2025. Outbreak data compiled through October 15, 2025.
(PDF)

S5 Table Provincial distribution of measles cases for 1999–2023 and multi-jurisdictional outbreak with standardized residuals. (PDF)

S1 Checklist PRISMA Scr Checklist. (PDF)
