# Supplementary material for: Epidemiological review on the resurgence of measles outbreaks in Canada during the post-elimination era: A scoping review
Source: PLOS Glob Public Health. 2026 Apr 13;6(4):e0006295. doi: 10.1371/journal.pgph.0006295 (PMC13075710; doi:10.1371/journal.pgph.0006295)
Supplement: S1 Appendix — (PDF) [file pgph.0006295.s001.pdf]

Meta-Analysis in Hospitalization

Articles	Year	Provinces	Health Territories	Total Cases*	Hospitalization
(Quebec MSSS, 2009)	2007	Quebec	Estrie, Montérégie, Mauricie et Centre-du-Québec, Laurentides, Montréal, Lanaudière, Bas-Saint-Laurent	94	11
(Armstrong et al., 2014)	2009	Ontario	Waterloo Public Health	6	1
(Quebec MSSS, 2012)	2011	Quebec	Bas-Saint-Laurent, Saguenay–Lac-Saint-Jean, Capitale-Nationale, Mauricie et Centre-du-Québec, Estrie, Montréal, Chaudière-Appalaches, Lanaudière, Laurentides, Montérégie	776	85
(Saskatchewan PHB, 2017)	2011	Saskatchewan	Five Hills, Prairie North, Prince Albert Parkland, Regina Qu'Appelle, Saskatoon, and Sun Country health regions	6	1
(Saskatchewan PHB, 2019)	2014	Saskatchewan	Unspecified	16	2
(Naus et al., 2015)	2014	British Columbia	Fraser East Health Authority	325	5
(Thomas et al., 2017)	2015	Ontario	Toronto PHU, Niagara PHU, York PHU, Halton PHU	18	2
(Panić and Gheorghe, 2016)	2015	Quebec	Lanaudière	159	14
(CMRSS, 2025b)	2025	Canada, 2024-2025	Alberta, British Columbia, Manitoba, New Brunswick, Northwest Territories, Nova Scotia, Ontario, Prince Edward Island, Quebec, Saskatchewan	5078	368

***Total cases:** Confirmed cases based on laboratory testing, clinical presentation, or epidemiological linkage.

Subgroup Analysis

A) Pre-2015 and 2015-2025

a. 2007-2015

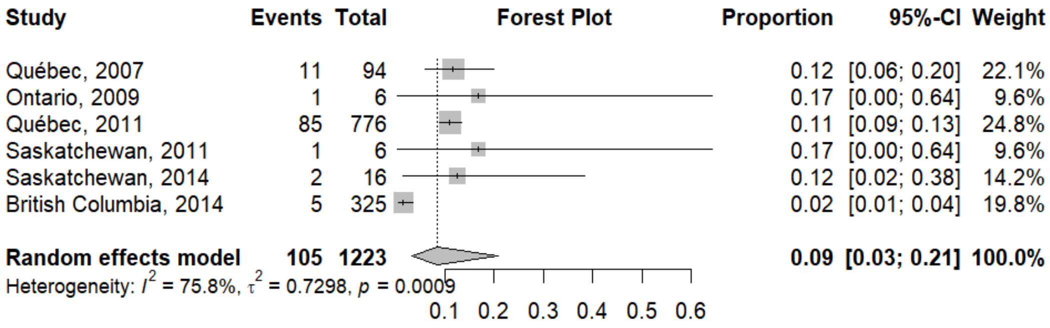

b. 2015-2025

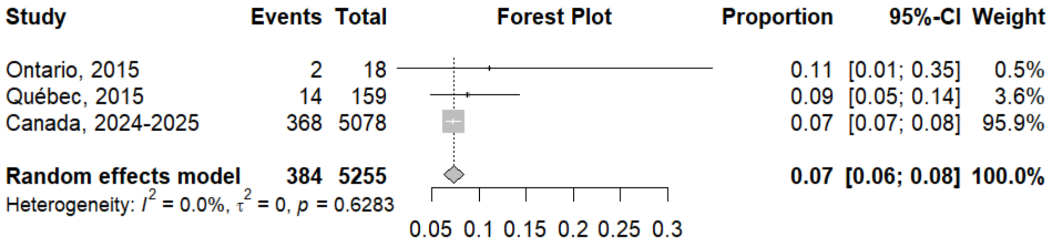

B) Total Confirmed Cases

a. <100

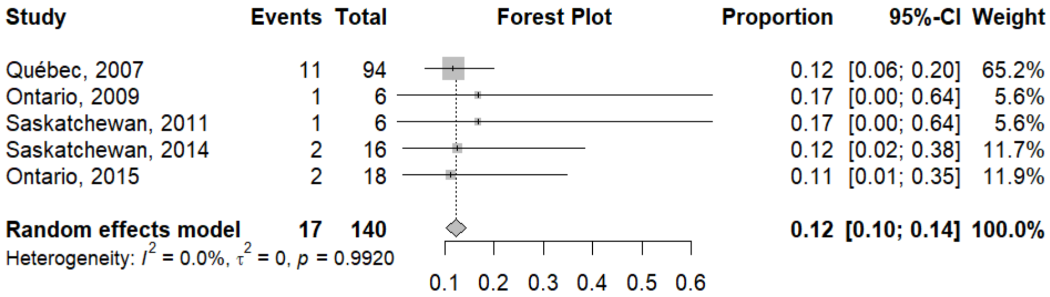

b. >100

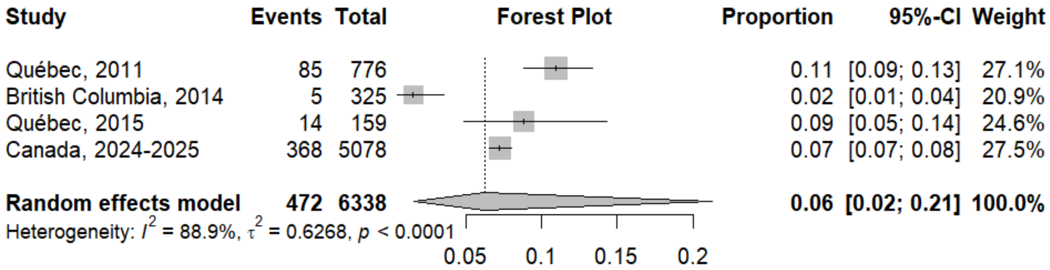

C) Study Weight

a. Low-Weight Study (>15%)

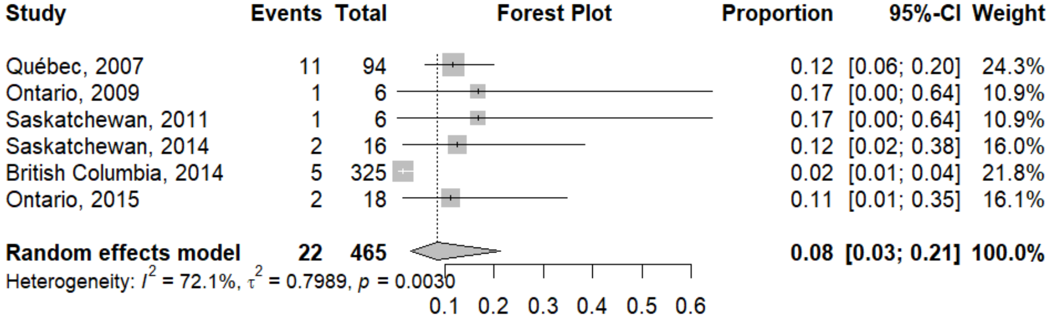

b. High-Weight Study (>15%)

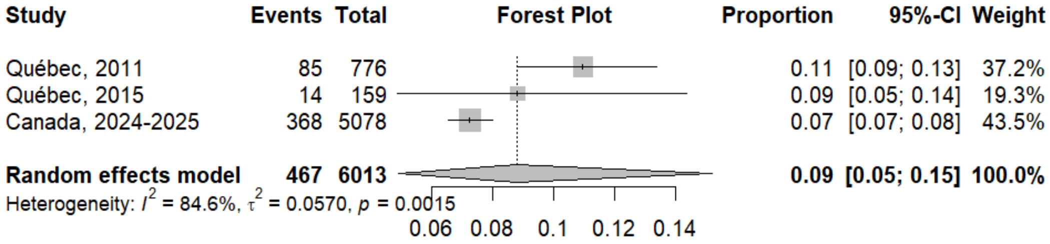

REFERENCES

Quebec MSSS, 2009:
https://msss.gouv.qc.ca/professionnels/documents/rougeole/eclosion_de_rougeole_2007.pdf
Armstrong et al., 2014: 14vol40_12-eng.pdf
Quebec MSSS, 2012:
https://msss.gouv.qc.ca/professionnels/documents/rougeole/rapport_final_eclosion_2011.pdf
Saskatchewan PHB, 2017:
https://publications.saskatchewan.ca/api/v1/products/91258/formats/108145/download
Saskatchewan PHB, 2019:
https://publications.saskatchewan.ca/api/v1/products/101170/formats/111793/download
Naus et al., 2015: ccdrv41i07a02-eng.pdf
Thomas et al., 2017: https://wwwnc.cdc.gov/eid/article/23/7/16-1145_article#;
Panić and Gheorghe, 2016: RAP_rougeole_2016.pdf
CMRSS, 2025b: https://health-infobase.canada.ca/measles-rubella/
