# Supplementary figures and images for: Epidemiological review on the resurgence of measles outbreaks in Canada during the post-elimination era: A scoping review
Source: PLOS Glob Public Health. 2026 Apr 13;6(4):e0006295. doi: 10.1371/journal.pgph.0006295 (PMC13075710; doi:10.1371/journal.pgph.0006295)

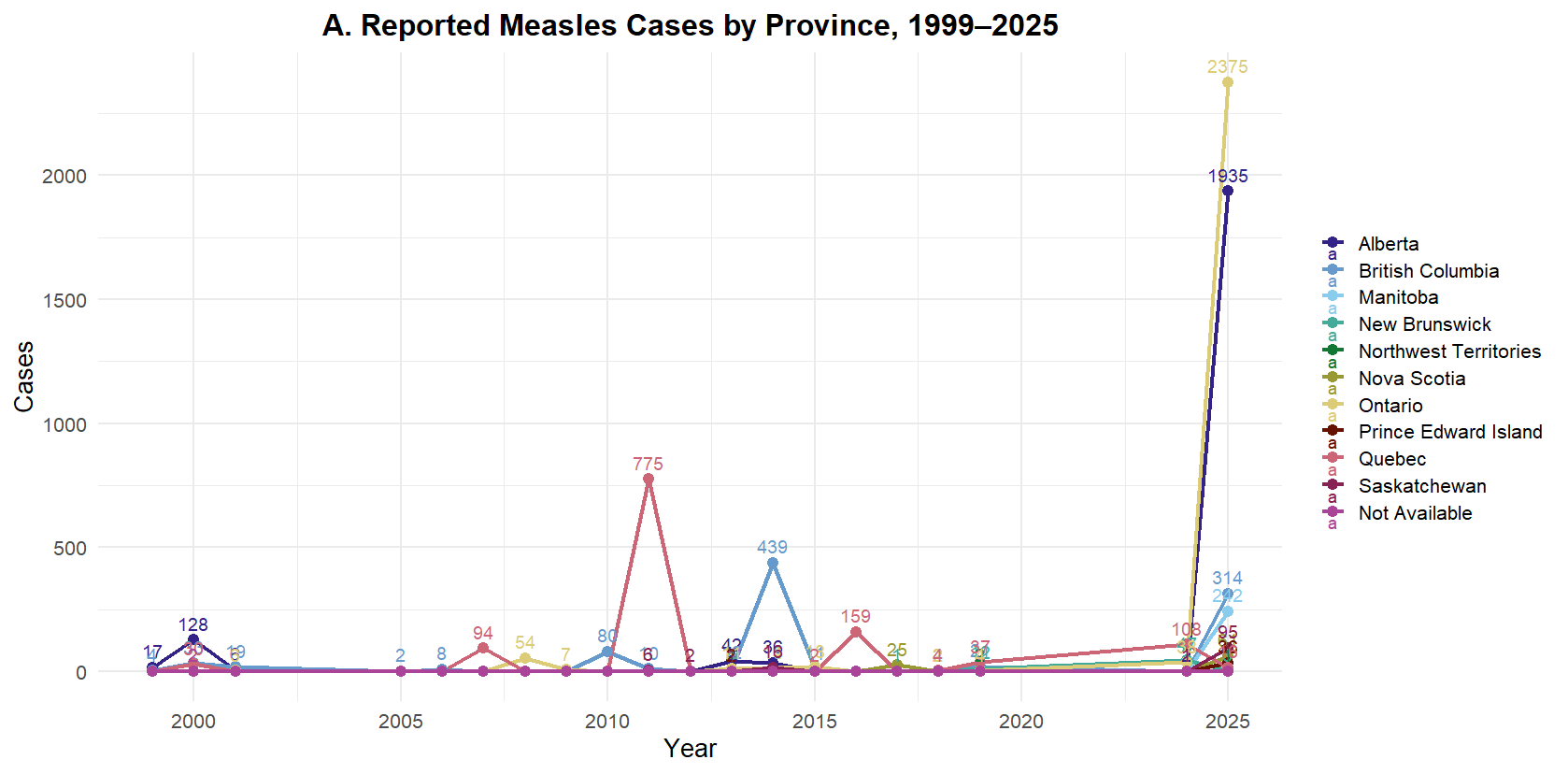

Supplement: S1 Fig — (TIFF) [file pgph.0006295.s002.tiff]
