# Supplementary material for: Epidemiological review on the resurgence of measles outbreaks in Canada during the post-elimination era: A scoping review
Source: PLOS Glob Public Health. 2026 Apr 13;6(4):e0006295. doi: 10.1371/journal.pgph.0006295 (PMC13075710; doi:10.1371/journal.pgph.0006295)
Supplement: S1 Table — Outbreak data compiled through October 15, 2025. (PDF) [file pgph.0006295.s003.pdf]

S1 Table. Summary of Measles Outbreaks in Canada, 1999-2025. Outbreak data compiled through October 15, 2025.

No.	Year	Provinces	Outbreak Period	Index Patient(s)	Mode of Transmission	Strain	Health Territories	Total Cases/ Incidence Rates	Case(s) MMR Vaccination Status				Reference(s)
									2D[a]	1D[b]	UV[c]	NS[d]	
1	1999	Alberta	N/A	The index case was unknown.	Imported from Europe. The outbreak occurred among unimmunized contacts.	N/A	N/A	0.58/100.000					AHW, 2001
2	1999	Alberta	3 generations	A mother and her 3 children returned from the Netherland	Imported from the Netherlands. The outbreak occurred among unimmunized members of a religious community.	D6 (MVi/Alberta.CAN/43.99/1)	N/A	17			17		King et al, 2004; Tipples et al, 2004
3	1999	British Columbia	Outbreak started on 30 July 1999 (2 Generations)	A Visitor from Holland who was infectious upon his arrival in Canada.	Imported from the Netherlands. The outbreak occurred among unimmunized members of a religious community.	N/A	South Fraser Valley	4	0	0	4	0	BCCDC, 2001 (a); King et al, 2004
4	2000	Alberta	The outbreak spanned 6 weeks (3 generations).	A 14-year-old girl visited Mazatlán, a tourist area in Mexico, during 10–24 March 2000.	Imported from Mexico. All cases were 3 generations of transmission traced back to the index case and happened in Canada.	D7 (MVs/Alberta.CAN/20.00/1)	N/A	6	0	0	6	0	King et al, 2004; Tipples et al, 2004
5	2000	Alberta	Outbreak lasted for 20 weeks and 10 generations and spread to British Columbia.	2 unimmunized siblings traveled to an affiliated religious community in Bolivia.	Imported from Bolivia. The index case attended a large social gathering in British Columbia on 13 June.	D6 (MVs/Alberta.CAN/23.00/1)	N/A	33					King et al, 2004; Tipples et al, 2004; BCCDC, 2001 (b);
		British Columbia					South Fraser Valley, Peace Liard, Vancouver	122 (1.0/100,000)					
6	2000	Quebec	The outbreak lasted 13 weeks and 6 generations.	The Index case was unknown.	Suspected imported from New York and Belgium. The outbreak occurred among semi-closed religious and unvaccinated communities in Quebec (population 2500)	D7 (MVs/Alberta.CAN/20.00/1)	N/A	30	100	150			King et al, 2004; Tipples et al, 2004
7	2000	N/A	N/A	7-month-old infant who had traveled to Pakistan and was hospitalized in Canada with undiagnosed measles.	Imported from Pakistan. The Index case infected a hospitalized 14-month-old child whose measles immunization had been delayed because of an illness.	N/A	N/A	2	0	0	2	0	King et al, 2004
8	2001	Alberta	N/A	The index case was unknown.	Imported from Germany	D5 (MVs/Calgary.CAN/20.01)	Calgary	3					King et al, 2004; Tipples et al, 2004; BCCDC 2001 (b);
9	2001	Alberta	N/A	The index case was unknown.	Imported from New Zealand	D5 (MVi/Calgary.CAN/40.01)	Calgary	3					King et al, 2004; Tipples et al, 2004; BCCDC 2001 (b);
10	2001	British Columbia	1 generation	The index case was initially an unrecognized imported index case of measles returning from an overseas Christmas-period holiday.	Imported from overseas (unknown country). Outbreaks happened among young teens	N/A	Fraser Valley	7 (0.6/100,000)					BCCDC, 2002
11	2001	British Columbia	N/A	The index case was unknown.	Imported from New Zealand	N/A	N/A	2					King et al, 2004; Tipples et al, 2004; BCCDC 2001 (b);
12	2001	British Columbia	N/A	The index case was unknown.	Imported from Pakistan.	N/A	N/A	2					King et al, 2004; Tipples et al, 2004; BCCDC 2001 (b);
13	2001	Ontario	N/A	The index case was unknown.	Imported from Singapore.	H1 (MVs/Hamilton.CAN/9.01)	Hamilton	3					King et al, 2004; Tipples et al, 2004; BCCDC 2001 (b);
14	2001	British Columbia	The outbreak spanned 24 days (2 generations).	15-year-old unvaccinated foreign-born student after a holiday trip to Korea.	Imported from Korea. The index case attended a school of more than 750 students and lived in a community who opposed immunization for philosophical reasons.	H1 (MVs/Vancouver.CAN/6.01/1)	Vancouver	8					King et al, 2004; Tipples et al, 2004; BCCDC 2001 (b);
15	2005	British Columbia	N/A	The index case was unknown.	Imported from the USA. The date of onset in the first case was consistent with the acquisition of the infection at an amusement park in California attended by people from all over the world.	N/A	Fraser South	2	0	0	2	0	BCCDC, 2006
16	2006	British Columbia	6 weeks	An adult male in his mid-40s with an unknown immunization history and no history of travel or contact with people experiencing a febrile rash.	Unknown exposure. Three subsequent cases occurred among children aged 4 years, 7 months, and 20 months.	D4 (circulating in Sudan, Lebanon and Nepal)	Interior Health Authority (Okanagan)	4	0	0	4	0	BCCDC, 2007
17	2006	British Columbia	N/A	A woman in her mid-30s with a travel history to a large amusement park in California.	Imported from the USA. A number of unimmunized children and adolescent contacts were exposed in a household setting. Three of the contacts developed measles-like illness.	D8 (circulating in Bangladesh, India and Nepal)	Fraser Health Authority (Fraser South)	4	0	0	3	1	BCCDC, 2007
18	2007	Quebec	April 19, 2007 - October 1, 2007 (25 weeks and 3 second generations (12-17 generations))	A 37-year-old male Canadian of Lebanese origin with unknown vaccination status and onset of rash on 19 April. The first case occurred in Montérégie on April 19.	The index case had traveled to Alberta (Canada) but had returned to Quebec 21 days before the rash onset, a delay that exceeded the usual incubation period. Before the index case patient became aware he had measles, he had been to crowded areas and used the public transportation system.	D4 (MVs/Quebec.CAN/16.07/1) closely related to another measles strain imported from Lebanon into the United States in 2003	Estrie	32	6	11	68	0	Quebec MSSS, 2009; Dallaire et al, 2009
							Montérégie	28					
							Mauricie et Centre-du-Québec	8					
							Laurentides	3					
							Montréal	16					
							Lanaudière	4					
							Bas-Saint-Laurent	3					
19	2008	Ontario	March - June 2008	The index case was unknown.	Suspected import-related because of common exposure to a major tourist destination in Toronto and the identification of measles genotype D8.	D8 (circulating in Southeast Asia and Eastern Mediterranean region)	Toronto	54					Wilson et al, 2015
20	2009	Ontario	Outbreak started on May 25, 2009 (2 Generations)	a 10-year-old unimmunized female had travelled to Walt Disney World, Florida, from May 3 to 10, 2009.	Imported from the USA. The index case transmitted measles to two unimmunized siblings of the case (9 and 6 year old), 39 year old female chaperoned a mall excursion for a community group attended by index case, a female that had been at the local shopping mall associated with the outbreak and her sibling, and probable case of an 11 year old female that develop measles after exposure to index case at basketball game/party.	D4 (identified in UK case, had been circulating in the south of England since February 2009)	Region of Waterloo Public Health	7*	2	2	3	0	Armstrong et al, 2014
21	2010						Okanagan	2	7	0	29	30	

No.	Year	Provinces	Outbreak Period	Index Patient(s)	Mode of Transmission	Strain	Health Territories	Total Cases/ Incidence Rates	Case(s) MMR Vaccination Status				Reference(s)
									2D[a]	1D[b]	UV[c]	NS[d]	
		British Columbia	The H1 outbreak spanned from 23 February to 26 May 2010. Onset dates ranged from March 9 through April 28, peaking April 6th.	Initial cases were in the Lower Mainland which spread throughout British Columbia. Two thirds of cases reported during the outbreak had no recognized source of infection, indicative of unrecognized and/or unreported cases.	Imported from China. Large outbreak following the Winter Olympic Games held in February, person-to-person transmission characterized by 100% genomic identity between cases.	H1 (MVi/BritishColumbia.CAN/12.10/1) identical to MV isolated in China	Thompson Cariboo Shuswap	12					BCCDC, 2011; Gardy et al, 2015
							Fraser East	1					
							Fraser North	12					
							Fraser South	11					
							Richmond	1					
							Vancouver	19					
							North Shore/Coast of Garibaldi	3					
							South Vancouver Island	2					
							Northwest	1					
							Northeast	16					
22	2011	British Columbia	Rash onset in February	Measles cases associated with a ski resort town located in Okanagan HSDA.	Infected one visitor from Vancouver Coastal Health Region, and 7 residents from person-to-person contact. 5 of the residents attended the same high school.	D4 (recorded in North America, South America, Europe, Russia, Japan and Australia)	Thompson Cariboo Shuswap	7	2	0	3	3	BCCDC, 2012;
							Vancouver	1					
23	2011	British Columbia	N/A	Unimmunized twin infants (<one year old) infected during travel to India	Imported from India. Does not resulted in any known transmission of measles in British Columbia	D8 (consistent with acquisition in India)	Fraser South	2	0	0	2	0	BCCDC, 2012;
24	2011	Quebec	January 8 to December 22, 2011. Sustained local transmission observed in April inside school settings and community. The majority of cases occurred between May 1 and July 30, 2011 (678 cases). There was a resurgence of transmission in September that was contained and quickly controlled.	Outbreak associated with 2 families traveled together in France, and a 39-year-old one-dose vaccinated high school staff who became ill 9 days after returning from a Caribbean country. Public health investigators suspect measles to have most likely been acquired in Canada at the Montreal airport.	Imported from France. 615 cases (79%) could not be considered protected.	D4 (circulating in Europe)	Bas-Saint-Laurent	1	76	7	95	187	Quebec MSSS, 2012; Billard et al, 2017; de Serres et al, 2012; de Serres et al, 2013
							Saguenay-Lac-Saint-Jean	2					
							Capitale-Nationale	31					
							Mauricie et Centre-du-Québec	538					
							Estrie	26					
							Montréal	15					
							Chaudière-Appalaches	5					
							Lanaudière	3					
							Laurentides	9					
							Montérégie	145					
25	2011	Saskatchewan	13 generations of transmission	3 cases acquired measles internationally, travel was reported to Europe and Philippines during the incubation period.	Imported from Europe and Philippines (3 cases). Other cases transmitted through household contact, social contact or contact with a case with international travel history.	B3 (3 cases), D8 (5 cases), D9 (4 cases)	Five Hills, Prairie North, Prince Albert Parkland, Regina Qu'Appelle, Saskatoon, and Sun Country health regions	6	2	2	1	4	Saskatchewan PHB, 2017
	2012							2					
	2013							1					
26	2013	Alberta	October 16, to November 25, 2013 (6 weeks with 4 generations)	Unvaccinated student at Coaldale Christian School exposed in the Netherland.	Imported from the Netherlands. Outbreak occurred in a religious community that does not participate in immunization programs.	D8 (MVs/Taunton.GBR/27.12)	South Health Zone	42					Shane et al, 2014; Alberta Health, 2015; Giddings and Sibbald, 2014
27	2013	British Columbia	January 31 to February 12, 2013 (2 weeks and 2 generations)	Index case had recent travel to Thailand.	Imported from Thailand. The secondary case was household contact.	D8 (MVi/Villupuram.Ind/03.072)	N/A	2					Shane et al, 2014;
28	2013	British Columbia	June 10 to June 26 (3 weeks and 2 generations)	Index case had recently traveled to New York City.	Suspected exposure at Vancouver International Airport.	D8 (MVs/Taunton.GBR/27.12)	N/A	3			1	2	Shane et al, 2014;
29	2013	British Columbia	June 24 to July 6 (2 weeks and 2 generations)	The index case was an unimmunized 25- to 29-year-old, without a history of travel.	Unknown exposure. Three secondary cases were associated with the index.	D8 (MVs/Taunton.GBR/27.12)	N/A	4	2	1	1		Shane et al, 2014;
30	2013	British Columbia	September 2 to September 16, 2013 (2 weeks and 2 generations)	The index case was an unimmunized infant (<1 year old), without history of travel.	Unknown exposure. Two secondary cases were associated with the index.	B3 (MVi/Harare.ZWE/38.09)	N/A	3			2	1	Shane et al, 2014;
31	2013	New Brunswick and Ontario	February 19 to March 21, 2013 (2 weeks and 2 generations)	The index case was unknown.	Imported from the UK. Two Canadians (1 from Ontario, 1 from New Brunswick) were exposed to this measles case at the resort. There were no secondary cases associated with the Ontario case, but 2 secondary cases from the New Brunswick case occurred among unimmunized families.	D8 (MVs/New Brunswick.CAN/7.13 and MVs/Ontario.CAN/8.13)	New Brunswick Health Region 2	4			3	1	Shane et al, 2014; OCMOH, 2014
32	2013	Ontario	February 24 to March 21, 2013 (4 weeks and 3 generations)	The index case was unknown.	An outbreak occurred in a Childcare center.	B3 (MVi/Harare.ZWE/38.09)	N/A	5	2		3		Shane et al, 2014;
33	2013	Ontario	June 8 to July 24, 2013 (9 weeks and 4 generations)	Co-index were unimmunized children aged 1 to 9 years old who traveled recently to British Columbia.	Outbreaks occurred in the health care setting.	D8 (MVs/Taunton.GBR/27.12)	N/A	6			3	3	Shane et al, 2014;
34	2013	Prince Edward Island	June 10 to June 22 (2 weeks and 2 generations)	Unimmunized young Canadian travelled to Europe (Austria, Italy, France)	Imported from Europe (Austria, Italy, and France). The secondary case was a sibling of the index case.	D8 (MVs/Taunton.GBR/27.12)	N/A	2			2		Shane et al, 2014;
35	2014	Alberta	4 days and 1 generation	The index case was unknown.	Unknown exposure. 3 cases presumed to have shared a common source of exposure based on their dates of rash onset.	B3 (MVi/Harare.ZWE/38.09)	N/A	3					Sherrard et al, 2015;
36	2014	Alberta	5 days and 1 generation	The index case was unknown.	Unknown exposure. 2 cases presumed to have shared a common source of exposure based on their dates of rash onset.	D8 (MVs/Taunton.GBR/27.12)	N/A	2					Sherrard et al, 2015;
37	2014	Alberta	27 days and 3 generations	Index case was unimmunized Canadian travelled to the Philippines	Imported from the Philippines. The secondary cases were import-related, epidemiologically linked cases in the same health unit.	B3	N/A	4					Sherrard et al, 2015;
38	2014	Alberta	53 days and 5 generations	Index case was two immigrants who travelled together from the Philippines	Imported from the Philippines.	B3 (MVi/Harare.ZWE/38.09)	N/A	9					Sherrard et al, 2015;
39	2014	Alberta	13 days and 2 generations	The index case was unknown.	Imported from India.	D8	N/A	2					Sherrard et al, 2015;

No.	Year	Provinces	Outbreak Period	Index Patient(s)	Mode of Transmission	Strain	Health Territories	Total Cases/ Incidence Rates	Case(s) MMR Vaccination Status				Reference(s)
									2D[a]	1D[b]	UV[c]	NS[d]	
40	2014	Alberta	Outbreak declared on April 29, 2014, and declared end on June 12, 2014 (45 days)	The index case was unknown.	Unknown exposure.	N/A	Calgary	9					Bandara et al, 2022;
41	2014	Alberta	Outbreak declared on April 29, 2014, and declared end on July 4, 2014 (67 days)	Index case was a child who flew to Edmonton from the Philippines via Vancouver	Imported from the Philippines. All passengers on board were at risk of infection.	N/A	Edmonton	7					Bandara et al, 2022;
42	2014	British Columbia	14 days and 2 generations	The index case was unknown.	Unknown exposure.	B3 (MVi/Harare.ZWE/38.09)	N/A	2					Sherrard et al, 2015;
43	2014	British Columbia	February 22 to June 9, 2014 (107 days and 9 generations)	The index case was unknown.	Suspected imported from the Netherlands. Subsequent spread occurred within a non-immunizing religious community primarily in a school-based setting.	D8 (MVs/Taunton.GBR/27.12)	Fraser East Health Authority	433	2	3	374	54	Sherrard et al, 2015; Naus et al, 2015; Deehan and Shane, 2014; BCCDC, 2015; Giddings and Sibbald, 2014
44	2014	British Columbia	18 days and 2 generations	The index case was unknown.	Unknown exposure. The secondary case was exposed in a health care setting.	B3 (MVi/Harare.ZWE/38.09)	N/A	2					Sherrard et al, 2015;
45	2014	British Columbia	9 days and 2 generations	The index case was unknown.	Unknown exposure. The secondary case was household contact.	B3 (MVi/Harare.ZWE/38.09)	N/A	2					Sherrard et al, 2015;
46	2014	Manitoba	15 days and 2 generations	The index case was unknown.	Unknown exposure. The secondary case was a close household contact of the index case.	B3 (MVi/Harare.ZWE/38.09)	N/A	2					Sherrard et al, 2015;
47	2014	Manitoba	21 days and 2 generations	The index case was unknown.	Unknown exposure. The secondary case was exposed in a healthcare setting.	B3 (MVi/Harare.ZWE/38.09)	N/A	2					Sherrard et al, 2015;
48	2014	Manitoba	13 days and 2 generations	The index case was unknown.	Imported from India. The secondary case had a weak epi-link to the index case.	D4	N/A	2					Sherrard et al, 2015;
49	2014	Manitoba	Spring 2014. 8 cases occurred between the beginning of March and the end of April 2014; the 9th case occurred in August (imported cases)	The index case was unknown.	Unknown exposure. The secondary case was a close household contact of the index case.	N/A	Winnipeg, Interlake-Eastern, Prairie Mountain, Northern RHA	9					Manitoba PHB, 2015;
50	2014	Ontario	26 days and 3 generations	Index case was unimmunized child travelled to the Philippines	Imported from the Philippines. The secondary cases were household and school contacts.	B3 (MVi/Harare.ZWE/38.09)	N/A	4					Sherrard et al, 2015;
51	2014	Ontario	12 days and 2 generations	Index case was immunized Canadian travelled to Thailand	Imported from Thailand. The secondary case was household contact.	B3	N/A	2					Sherrard et al, 2015;
52	2014	Ontario	19 days and 2 generations	Index case was Canadian travelled to the Philippines and China	Imported from the Philippines. The two secondary cases were exposed to measles in a health care setting.	B3 (MVi/Harare.ZWE/38.09)	N/A	3					Sherrard et al, 2015;
53	2014	Ontario	12 days and 1 generation	Index case visitor from China linked to ongoing outbreak in China.	Imported from China. All five cases were epidemiologically linked to this one visitor.	D9	N/A	5					Sherrard et al, 2015;
54	2014	Saskatchewan	32 days and 3 generations	Index case was unimmunized Canadian travelled to the Philippines	Imported from the Philippines. Subsequent spread was reported among contacts in a variety of settings. Transmission is believed to have occurred in health care facilities (6 cases). Three other cases were exposed either by household contact or social contact.	B3 (MVi/Harare.ZWE/38.09)	Regina Qu'Appelle, Saskatoon, and Sun Country health regions	10	1	2	12	1	Sherrard et al, 2015; Saskatchewan PHB, 2017; Saskatchewan PHB, 2019
55	2014	Saskatchewan	9 days and 2 generations	The index case was unknown.	Unknown exposure. The reported cases are presumed to have shared a common source of exposure. Transmission is believed to have occurred in health care facilities (6 cases). Three other cases were exposed either by household contact or social contact.	D8 (MVs/Taunton.GBR/27.12)	Regina Qu'Appelle, Saskatoon, and Sun Country health regions	6	1	2	12	1	Sherrard et al, 2015; Saskatchewan PHB, 2017; Saskatchewan PHB, 2019
56	2015	British Columbia	19 days and 2 generations	Two co-indexes were reported among Canadians who were exposed during travel to China.	Imported from China, the co-indexes were communicable during the return flight to Canada. Subsequent spread to passengers and individuals linked to the flight.	H1	N/A	11					Sherrard et al, 2016;
57	2015	Ontario	Outbreak declared on January 25, 2015, and declared end on February 17, 2015 (24 days)	The index case was unknown.	These cases had no epidemiologic link to each other, or to a known case. However, based on dates of rash onset and genotype results, it is presumed that they shared a common source of exposure.	D4	Toronto PHU	10	1	3	3	3	Thomas et al, 2017; Ramsay et al, 2019; Ontario AHPP, 2017; Sherrard et al, 2016
				Index case was Canadian travelled to Toronto during outbreak	The presumed index case shared a common source of exposure with the Toronto case. Only one of the cases resulted in secondary spread (to five household contacts).		Niagara PHU	6			6		
				2 co-indexes with no history of travel.	These cases had no epidemiologic link to each other, or to a known case. However, based on dates of rash onset and genotype results, it is presumed that they shared a common source of exposure.		York PHU	1				1	
							Halton PHU	1	1				
58	2015	Quebec	14 days and 2 generations	The index case had a history of travel to India.	Imported from India. One secondary case was reported, who was exposed to measles in a health-care setting.	D8	N/A	2					Sherrard et al, 2016;
59	2016	Quebec	72 days and 6 generations. The first case was reported on March 20, 2016.	The index case was an unvaccinated traveler exposed to measles during travel to a theme park in California, USA, from December 21, 2014, to January 9, 2015.	Imported from the USA. Subsequent spread occurred in a non-immunizing religious community.	B3 (MVi/Harare.ZWE/38.09)	Lanaudière	159					Sherrard et al, 2016; Quebec CISSS, 2016;
60	2017	New Brunswick	N/A	The index case was unknown.	Related to the Nova Scotia outbreak.	N/A	New Brunswick Health Region 3	1					OCMOH, 2018;
61	2017	Nova Scotia	The first case was reported in January 2017. Additional cases still identified in April 2017.	The index case was a student at Hebbville Academy in South Shore.	Unknown exposure. Transmission occurred person-to-person in high school and community settings.	N/A	Nova Scotia Central	15					NSDHW, 2018; The Advance Canada, 2017;
				The index case was unknown.	Transmission occurred person-to-person in high school and community settings.	N/A	Nova Scotia Western	7					

No.	Year	Provinces	Outbreak Period	Index Patient(s)	Mode of Transmission	Strain	Health Territories	Total Cases/ Incidence Rates	Case(s) MMR Vaccination Status				Reference(s)
									2D[a]	1D[b]	UV[c]	NS[d]	
62	2017	Nova Scotia	The first case reported on February 9, 2017.	The index case was unknown.	All 3 of the cases are linked, and the people affected are known to each other. They all live in the Halifax area but traveled within the province during the incubation period.	N/A	Halifax	3					Canada CBC, 2017;
63	2018	Alberta	Index case rash onset reported on May 13, 2018.	The index case was Canadian travelled to India during exposure period	Imported from India. One secondary case, a close contact of the index case.	D8	N/A	2			1	1	Coulby et al, 2020;
64	2018	British Columbia	Index case rash onset reported on August 26, 2018.	The index case was Canadian exposed to international traveler at Canadian port	Suspected exposure to international measles case in Canadian Port. The index case did not report travel outside of Canada. One secondary case, a contact of the index case.	D8 (MVs/Osaka.JPN/29.15/)	N/A	2	2				Coulby et al, 2020;
65	2018	British Columbia	Index case rash onset reported on November 4, 2018.	The index case was Canadian travelled to Philippines during exposure period	Imported from the Philippines. One secondary case, a close contact of the index case.	B3	N/A	2	1			1	Coulby et al, 2020;
66	2018	Manitoba	Index case rash onset reported on September 9, 2018.	The index case was Canadian travelled to Southeast Asia during exposure period	Imported from Southeast Asia. One secondary case, a close contact of the index case.	D8 (MVs/Samut Sakhon.THA/49.16)	N/A	2	1		1		Coulby et al, 2020;
67	2018	Ontario	Index case rash onset reported on June 3, 2018.	The index case was Canadian travelled to Ukraine during exposure period	Imported from Ukraine. One secondary case, a close contact of the index case.	D8 (MVs/Gir Somnath.IND/42.16/)	N/A	2			1	1	Coulby et al, 2020;
68	2018	Quebec	Index case rash onset reported on April 15, 2018.	The index case was Canadian travelled to Romania during exposure period	Imported from Romania. Three secondary cases among family contacts of the index case.	B3 (MVs/Dublin.IRL/8.16/)	N/A	4			4		Coulby et al, 2020;
69	2019	Alberta	Index case rash onset reported on May 18, 2019 (2 generations).	The index case was Canadian travelled to Viet Nam and Thailand during exposure period	Imported from Viet Nam and/or Thailand. The secondary case was workplace contact.	D8 (MVs/GirSomnath.IND/42.16,468 3)	N/A	2				2	Coulby et al, 2021;
70	2019	British Columbia	Index case rash onset reported on February 2, 2019, and ended on April 3, 2019 (2 generations).	The 3 co-index cases were Canadian children travelled to Viet Nam during exposure period	Imported from Viet Nam. Ten subsequent cases were reported; primary exposure occurred in two schools.	D8 (MVs/GirSomnath.IND/42.16,468 3)	Vancouver Coastal Health	13		4	9		Coulby et al, 2021; BCCDC, 2019
71	2019	British Columbia	Index case rash onset reported on March 9, 2019 (2 generations).	The index case was Canadian travelled to USA during exposure period	Imported from the USA. The secondary case was a family contact of the index case.	D8 (MVs/Dagon Seikkan.MMR/5.18,5551)	Interior Health Authority region	2	1		1		Coulby et al, 2021; BCCDC, 2019
72	2019	British Columbia	Index case rash onset reported on March 9, 2019 (2 generations).	The index case was Canadian travelled to Viet Nam during exposure period	Imported from Viet Nam. The secondary case was a family contact of the index case. Four subsequent cases compatible with acquisition in the earlier Vancouver school-related outbreak.	D8 (MVs/GirSomnath.IND/42.16,468 3)	Vancouver Island Health Authority	6		1	1		Coulby et al, 2021; BCCDC, 2019
73	2019	New Brunswick	Outbreaks occurred from April 25 to July 28 (3 generations).	The index case was Canadian travelled to various country in Europe during exposure period	Imported from Europe. The secondary case was a healthcare contact of the index case; 10 tertiary cases followed exposures in a school and in the community.	D8 (MVs/GirSomnath.IND/42.16,468 3)	New Brunswick Health Region 2	12	9	1	2		Coulby et al, 2021; OCMOH, 2020;
74	2019	Northwest Territories	Index case rash onset reported on February 16, 2019 (2 generations). A second case developed measles, showing symptoms on February 28, 2019.	The index case was unvaccinated Canadian child travelled to Philippines during exposure period	Imported from the Philippines. The secondary case was a contact of the index case.	B3 (MVi/Marikina City.PHL/10.18, 5306)	Inuvik	2		1	1		Coulby et al, 2021; Cabin radio, 2019;
75	2019	Ontario	Index case rash onset reported on March 23, 2019 (2 generations).	The index case was Canadian travelled to Bangladesh during exposure period	Imported from Bangladesh. The secondary case was a household contact of the index case.	B3	N/A	2		1	1		Coulby et al, 2021;
76	2019	Ontario	Index case rash onset reported on June 1, 2019 (2 generations).	The index case did not report travel outside of Canada.	Unknown exposure. The secondary case was household contact.	D8 (MVs/GirSomnath.IND/42.16,468 3)	N/A	2				2	Coulby et al, 2021;
77	2019	Quebec	Index case rash onset reported on June 15, 2019.	The index case was Canadian born before 1970 travelled to the USA during heightened measles activity in the USA.	Suspected importation from the USA. Several generations of transmission were linked to a shopping mall and a non-vaccinating community in the Montréal area.	D8 (MVs/GirSomnath.IND/42.16,468 3)	Laurentides and Laval	34		2	32		Coulby et al, 2021; Quebec MSSS, 2019
78	2019	Quebec	Index case rash onset reported on May 4, 2019 (2 generations).	The index case was Canadian travelled to France during exposure period	Imported from France. Two secondary cases were contacts of the index case.	B3	Montréal	3	1		2		Coulby et al, 2021; Quebec MSSS, 2019
79	2024	Quebec	The outbreak lasted over three months from January 28 to May 4, 2024, and was deemed over on June 5, 2024.	The index case was a student attending the school while being contagious.	Most cases (n= 43) were acquired in Quebec, with the others occurring outside the country. Healthcare facilities, childcare settings, and a large public event were the main transmission sites.	N/A	Montréal (18), Montérégie (11), Laval (5), Laurentides, Mauricie et Centre-du-Québec, Lanaudière, and Estrie	51					Quebec MSSS, 2024;
80	2024	New Brunswick	The outbreak began in New Brunswick in October 2024 and continued to spread in several jurisdictions (October 2024 - 31 December 2024).	The index case was when a traveler arrived in New Brunswick from the Philippines and stopped in Vancouver and Toronto. Related to the New Brunswick outbreak.	Imported from the Philippines. The index case attended a large gathering for Mennonite communities in New Brunswick.	B3 (54 cases); D8 (26 cases)	N/A	47	12	14	97	23	MRWMR, 2025a
		Ontario	The outbreak was first reported in October 2024		Transmission linked to unvaccinated individuals in Mennonite communities in New Brunswick, with exposures at churches and school.		Grand Erie and Southwestern PHU	38					
		Alberta	The outbreak was first reported in October 2024		Secondary cases from person-to-person contact.		N/A	2					
		British Columbia	The outbreak occurred between October 2024 and May 24, 2025				N/A	1					
		Saskatchewan	The outbreak occurred between October 2024 and May 24, 2025				N/A	1					
		Quebec	The outbreak began in December 2024				Laurentides	57					
	2025	New Brunswick	The outbreak began in New Brunswick in October 2024 and continued to spread in several jurisdictions.	Related to the New Brunswick outbreak.	Secondary cases from person-to-person contact.	B3 (35 cases); D8 (1320 cases); Pending (939 cases); Unknown (2575)	New Brunswick health Region 2	16	232	124	4,448	256	MRWMR, 2025b (Data from December 29, 2024 – October 15 (41 weeks))

No.	Year	Provinces	Outbreak Period	Index Patient(s)	Mode of Transmission	Strain	Health Territories	Total Cases/ Incidence Rates	Case(s) MMR Vaccination Status				Reference(s)
									2D[a]	1D[b]	UV[c]	NS[d]	
		Alberta	The outbreak was first reported in October 2024				North, South, Edmonton, Calgary, and Central Zone	1935					
		Ontario	The outbreak was first reported in October 2024				Waterloo HU, Windsor-Essex County HU, Chatham-Kent HU, Middlesex-London UH, North Bay Parry Sound District HU, Grey Bruce, Haliburton Kawartha Northumberland Peterborough HU, Halton Regional HU, Lambton HU, Niagara Regional Area HU, Peel Regional HU, City of Toronto HU, Wellington-Dufferin-Guelph HU, The District of Algoma HU, Southwestern HU, Grand Erie PHU, Huron Perth HU	2375					
		Saskatchewan	The outbreak occurred between October 2024 and May 24, 2025				North Central East, Southwest, Saskatoon Zone	95					
		Nova Scotia	The outbreak was first reported on May 24, 2025		No secondary cases reported.		Calgary, Central, Halifax and Northern Zone	61					
		Manitoba	The outbreak was first reported in February 2025		The exposure sites were the Winnipeg Jazz Orchestra event at the Franco-Manitobain cultural center.		Southern, Interlake-Eastern, Winnipeg-Churchill Health Region	242					
		British Columbia	The outbreak occurred between October 2024 and May 24, 2025	The 2nd index case was a traveler returning from a trip to Southeast Asia and residing in Fraser Health Region, arriving in Vancouver on February 11, 2025	Imported from Southeast Asia. Secondary cases from person-to-person contact.		Northern HA, Interior HA, Fraser HA, Island HA, Vancouver Coastal HA	314					
		Northwest Territories	The outbreak was first reported on May 4, 2025	A student from Yellowknife's Catholic School was exposed to measles during the school day.	The exposure sites were Yellowknife's Catholic school, Weledeh Catholic School and École St. Patrick High School. No secondary cases reported.		District of Algoma Health Unit	1					
		Prince Edward Island	The outbreak was first reported on May 4, 2025	2 co-indexes with history of travel within Canada measles zone, tested positive in April 2025.	Related to New Brunswick outbreak. 2 unrelated new cases of Measles in the province, linked to various public exposure sites.		Prince Edward Island	3					
		Quebec	The outbreak began in December 2024	The 2nd index case was an unvaccinated individual who had attended a KISS-themed hockey game on March 3, 2025	Secondary cases from person-to-person contact.		Montréal, Montérégie, Laval, Laurentides	36					

*(1 probable case)
2D[a] : 2 or more recorded doses of MMR Vaccine.
1D[b] : 1 recorded doses of MMR Vaccine.
UV[c] : Unvaccinated with MMR Vaccine.
NS[d] : Unknown record of MMR Vaccine.
N/A : No data.
