# Supplementary material for: Epidemiological review on the resurgence of measles outbreaks in Canada during the post-elimination era: A scoping review
Source: PLOS Glob Public Health. 2026 Apr 13;6(4):e0006295. doi: 10.1371/journal.pgph.0006295 (PMC13075710; doi:10.1371/journal.pgph.0006295)
Supplement: S2 Table — Outbreak data compiled through October 15, 2025. (PDF) [file pgph.0006295.s004.pdf]

S2 Table. Characteristics of Measles Outbreaks in Canada by Year, 1999–2025. Outbreak data compiled through October 15, 2025.

	TOTAL	(%)	99	‘00	‘01	‘05	‘06	‘07	‘08	‘09	‘10	‘11	‘12	‘13	‘14	‘15	‘16	‘17	‘18	‘19	‘24	‘25
**Diagnosis Type[a]**																						
**Total Case**	**7455**		21	193	28	2	8	94	54	7	80	791	2	72	520	31	159	26	14	78	197	5078
Laboratory Confirmed	925	12.4%	4	24	0	1	5	50	0	3	6	301	0	4	68	0	0	0	0	2	50	407
Epi-Linked	5111	68.6%	0	6	0	1	3	44	0	3	0	431	0	7	322	0	0	0	0	4	1	4289
Clinical Cases*	503	6.7%	0	0	0	0	0	0	0	0	0	58	0	0	110	0	0	0	0	0	0	335
Unknown	916	12.3%	17	163	28	0	0	0	54	1	74	1	2	61	20	31	159	26	14	72	146	47
**Vaccination Status[b]**	**6149**																					
2 doses	431	7.0%	0	100	0	0	0	6	0	2	7	80	0	4	4	2	0	0	4	11	12	200
1 dose	314	5.1%	0	150	0	0	0	11	0	2	0	9	0	1	7	3	0	0	0	10	14	109
Unvaccinated	4898	79.7%	21	8	0	2	7	68	0	3	29	101	0	15	398	9	0	0	7	49	97	4096
Unknown	506	8.2%	0	0	0	0	1	0	0	0	30	194	0	7	56	4	0	0	3	4	23	185
**Sex[c]**	**3249**																					
Male	1682	51.8%	0	0	0	0	0	0	0	0	42	11	0	0	239	8	69	0	0	0	82	1236
Female	1567	48.2%	0	0	0	0	0	0	0	0	38	8	0	0	235	10	85	0	0	0	64	1138
**Age group (years)[d]**	**6195**																					
Age <1	217	3.5%	0	0	0	0	0	0	0	0	9	24	0	1	15	0	3	0	0	0	11	157
Age 1-4	1230	19.9%	0	0	0	0	0	0	0	0	8	63	0	7	75	0	14	0	0	0	24	1041
Age 5-9	963	15.5%	0	0	0	0	0	0	0	0	7	61	0	4	129	0	35	0	0	0	44	688
Age 10-14	1950	31.5%	0	0	0	0	0	0	0	0	3	255	0	1	176	0	51	0	0	0	0	1464
Age 15-19	367	5.9%	0	0	0	0	0	0	0	0	7	226	0	1	61	0	31	0	0	0	0	41
Age 20-29	641	10.3%	0	0	0	0	0	0	0	0	14	45	0	4	14	1	18	0	0	0	65	486
Age 30-39	638	10.3%	0	0	0	0	0	0	0	0	20	56	0	6	1	1	5	0	0	0	0	549
Age >40	189	3.1%	0	0	0	0	0	0	0	0	12	14	0	0	3	0	2	0	0	0	2	156
**Importation Status[f]**	**84**																					
Imported	57	67.9%	3	4	7	1	1	0	1	1	1	3	0	5	12	2	1	0	5	8	1	1
Non-Imported	8	9.5%	0	0	0	0	1	1	0	0	0	1	0	1	0	1	0	0	0	0	2	1
Unknown exposure	19	22.6%	0	0	0	0	0	0	0	0	0	0	0	3	9	1	0	3	1	2	0	0

Diagnosis Type[a] : 53 outbreaks lack information on diagnosis type.
Vaccination Status[b] : 46 outbreaks lack information on vaccination status.
Sex[c] : 76 outbreaks do not include sex information.
Age group (years)[d] : 69 outbreaks are missing age group categorization.
Outbreak Size[e] : The 2015 Ontario outbreaks include both household and community transmission within certain regions (n=2)
Importation Status[f] :

- 2024 multiregional outbreaks involve regions with both imported and locally transmitted cases (n=2).
- 2025 multiregional outbreaks involve regions with both imported and locally transmitted cases (n=2).
- 2015 Ontario outbreaks include regions classified as non-imported or unknown exposure, suspected to be imported without clear evidence (n=2).

Clinical Cases* : Cases meeting clinical criteria but without epidemiologic links.
