# Supplementary material for: Epidemiological review on the resurgence of measles outbreaks in Canada during the post-elimination era: A scoping review
Source: PLOS Glob Public Health. 2026 Apr 13;6(4):e0006295. doi: 10.1371/journal.pgph.0006295 (PMC13075710; doi:10.1371/journal.pgph.0006295)
Supplement: S3 Table — (PDF) [file pgph.0006295.s005.pdf]

S3 Table. Age Group Distribution of Measles Cases and Standardized Residuals.

Age Group	Observed	Expected	Standardized Residual
< 1	217	774.375	-21.33
1-4	1203	774.375	16.63
5-9	963	774.375	7.39
10-14	1950	774.375	45.39
15-19	367	774.375	-15.55
20-29	641	774.375	-5.01
30-39	638	774.375	-5.12
> 40	189	774.375	-22.40

$X^2 = 3186.7$, df = 7, p-value < 2.2e-16
