# Supplementary material for: Epidemiological review on the resurgence of measles outbreaks in Canada during the post-elimination era: A scoping review
Source: PLOS Glob Public Health. 2026 Apr 13;6(4):e0006295. doi: 10.1371/journal.pgph.0006295 (PMC13075710; doi:10.1371/journal.pgph.0006295)
Supplement: S4 Table — Outbreak data compiled through October 15, 2025. (PDF) [file pgph.0006295.s006.pdf]

S4 Table. Provincial Distribution of Measles Cases in Canada by Year, 1999-2025. Outbreak data compiled through October 15, 2025

Provinces	Total	(%)	99	'00	'01	'05	'06	'07	'08	'09	'10	'11	'12	'13	'14	'15	'16	'17	'18	'19	'24	'25
**Alberta**	2170	29.1%	17 (81.0%)	128 (66.3%)	6 (21.4%)	0	0	0	0	0	0	0	0	42 (58.3%)	36 (6.9%)	0	0	0	2 (14.3%)	2 (2.6%)	2 (1.0%)	1935 (38.1%)
**British Columbia**	958	12.9%	4 (19.0%)	33 (17.1%)	19 (67.9%)	2 (100.0%)	8 (100.0%)	0	0	0	80 (100.0%)	10 (1.3%)	0	12 (16.7%)	439 (84.4%)	11 (35.5%)	0	0	4 (28.6%)	21 (26.9%)	1 (0.5%)	314 (6.2%)
**Manitoba**	259	3.5%	0	0	0	0	0	0	0	0	0	0	0	0	15 (2.9%)	0	0	0	2 (14.3%)	0	0	242 (4.8%)
**New Brunswick**	67	0.9%	0	0	0	0	0	0	0	0	0	0	0	4 (5.6%)	0	0	0	1 (3.8%)	0	12 (15.4%)	47 (23.9%)	3 (0.06%)
**Northwest Territories**	3	0.04%	0	0	0	0	0	0	0	0	0	0	0	0	0	0	0	0	0	2 (2.6%)	0	1 (0.02%)
**Nova Scotia**	86	1.2%	0	0	0	0	0	0	0	0	0	0	0	0	0	0	0	25 (96.2%)	0	0	0	61 (1.2%)
**Ontario**	2526	33.9%	0	0	3 (10.7%)	0	0	0	54 (100.0%)	7 (100.0%)	0	0	0	11 (15.3%)	14 (2.7%)	18 (58.1%)	0	0	2 (14.3%)	4 (5.1%)	38 (19.3%)	2375 (46.8%)
**Prince Edward Island**	38	0.5%	0	0	0	0	0	0	0	0	0	0	0	2 (2.8%)	0	0	0	0	0	0	0	36 (0.7%)
**Quebec**	1225	16.4%	0	30 (15.5%)	0	0	0	94 (100.0%)	0	0	0	775 (98.0%)	0	0	0	2 (6.5%)	159 (100.0%)	0	4 (28.6%)	37 (47.4%)	108 (54.8%)	16 (0.3%)
**Saskatchewan**	121	1.6%	0	0	0	0	0	0	0	0	0	6 (0.8%)	2 (100.0%)	1 (1.4%)	16 (3.1%)	0	0	0	0	0	1 (0.5%)	95 (1.9%)
**Unknown**	2	0.03%	0	2 (1.0%)	0	0	0	0	0	0	0	0	0	0	0	0	0	0	0	0	0	0
	**7455**		**21**	**193**	**28**	**2**	**8**	**94**	**54**	**7**	**80**	**791**	**2**	**72**	**520**	**31**	**159**	**26**	**14**	**78**	**197**	**5078**
