# Supplementary material for: Epidemiological review on the resurgence of measles outbreaks in Canada during the post-elimination era: A scoping review
Source: PLOS Glob Public Health. 2026 Apr 13;6(4):e0006295. doi: 10.1371/journal.pgph.0006295 (PMC13075710; doi:10.1371/journal.pgph.0006295)
Supplement: S5 Table — (PDF) [file pgph.0006295.s007.pdf]

S5 Table. Provincial Distribution of Measles Cases for 1999-2023 and Multi-Jurisdictional Outbreak with Standardized Residuals.

Province	Observed	Population Proportion	Standardized Residual
1999-2023 Outbreaks			
Alberta	233	0.1112	-0.56
British Columbia	634	0.1323	21.99
Manitoba	17	0.0363	-7.09
New Brunswick	17	0.0219	-4.47
Northwest Territories	2	0.0012	-0.37
Nova Scotia	25	0.0272	-4.49
Ontario	113	0.3839	-31.77
Prince Edward Island	2	0.042	-2.36
Quebec	1101	0.2340	30.10
Saskatchewan	25	0.0308	-5.20
Nunavut	0	0.0010	-1.47
Yukon	0	0.0010	-1.55
Newfoundland and Labrador	0	0.0149	-5.73
Multi-jurisdictional Outbreak			
Alberta	1937	0.1152	57.33
British Columbia	315	0.1352	-16.03
Manitoba	242	0.0363	3.72
New Brunswick	50	0.0210	-5.84
Northwest Territories	1	0.0011	-1.99
Nova Scotia	61	0.0262	-6.66
Ontario	2413	0.3845	10.89
Prince Edward Island	36	0.0042	2.95

Quebec	124	0.2298	-35.61
Saskatchewan	96	0.0306	-5.23
Nunavut	0	0.0010	-230
Yukon	0	0.0011	-2.41
Newfoundland and Labrador	0	0.0138	-8.59

1999-2023 outbreaks: χ^2 = 1898.8, df = 12, p-value = 0.0004998

Multi-jurisdictional outbreak: χ^2 = 4392.9, df = 12, p-value = 0.0004998
